# Volatile Compounds Profiling of Fresh *R. alba* L. Blossom by Headspace—Solid Phase Microextraction and Gas Chromatography

**DOI:** 10.3390/molecules30153102

**Published:** 2025-07-24

**Authors:** Daniela Antonova-Nedeltcheva, Ana Dobreva, Kamelia Gechovska, Liudmil Antonov

**Affiliations:** 1 Institute of Organic Chemistry with Centre of Phytochemistry, Bulgarian Academy of Sciences, 1113 Sofia, Bulgaria; kamelia.gechovska@orgchm.bas.bg; 2 Institute for Roses and Aromatic Plants, Agricultural Academy, 6100 Kazanlak, Bulgaria; anadobreva@abv.bg; 3 Institute of Electronics, Bulgarian Academy of Sciences, 1784 Sofia, Bulgaria

**Keywords:** *Rosa alba* L., solid-phase microextraction (SPME), gas chromatography-mass spectrometry (GC/MS), aroma profile, volatile organic compounds (VOCs), DVB/CAR/PDMS

## Abstract

The white oil-bearing rose (*R. alba* L.) is the second of the industrially important rose species for Bulgarian rose cultivation and essential oil production. In recent years, the interest in white oil-bearing rose has increased, following the worldwide trend for searching for new aromatic alternatives. Therefore, the purpose of the current research is to evaluate the volatile compounds profile of fresh *R. alba* L. flowers using headspace solid-phase microextraction (HS-SPME) and gas chromatography-mass spectrometry (GC/MS). More than 75 individual compounds were identified and quantified using HS-SPME-GC/MS. The study revealed that the aroma-bearing fraction of rose volatiles consists mainly of monoterpene alcohols; 2-phenylethanol was the most abundant component (8.4–33.9%), followed by geraniol (12.8–32.5%) and citronellol + nerol (17.7–26.5%). Linalool, α-pinene, β-myrcene, and rose oxides were also observed in low concentrations. The stearopten fraction in the HS phase was observed in low concentration, with main representatives nonadecane + nonadecene, heptadecane, heneicosane, and tricosane. The HS-GC profile of the *R. alba* fresh flowers shows distinct differences in relative abundance of the components between the two studied clones of the population, as well as between volatiles in petals and in the whole blossom. The absence of some undesirable components, such as allergenic and potentially carcinogenic methyl eugenol in fresh *R. alba* blossom, makes white oil-bearing rose a promising alternative to *R. damascena* in perfumery, natural cosmetics, and aromatherapy.

## 1. Introduction

Nowadays, *Rosa alba* L. is industrially cultivated for essential oil production only in Bulgaria, where it is the second of commercial importance only after *R. damascena* Mill. [1,2]. The revival in the interest in white oil-bearing rose is in line with the recent worldwide tendencies for searching for new aromatic alternatives [1]. The plant has been rediscovered since it possesses advantages over the highly valued Damask rose, such as increased tolerance to diseases and unfavorable climatic conditions [3]. The composition of the *R. alba* L. essential oil is very close to that of *R. damascena* and demonstrates many beneficial properties for perfumery, cosmetics, and medical applications, with a very low toxic potential [2,4,5]. This fact is due to the absence of some undesirable components, such as potentially allergenic methyl eugenol [1].

The industrial importance of oil-bearing roses is based on the extraction of the highly valued rose aroma from the rose flowers by a variety of methods (water steam distillation, solvent extraction, extraction with liquified gases, etc.). There is no doubt that the produced extracts (rose oil, rose concrete/absolute, rose sub/supercritical extracts) have aroma profiles different from the authentic one of the living flowers. This is caused by the niceties of the used technological procedures, and the best way to evaluate the changes in the aroma profile is to compare it with the aroma composition of the fresh, unprocessed flowers. Different parts of the flower emit different volatiles. Bearing in mind the high economic importance of the rose essential oil, it is valuable to reveal which volatile organic compounds (VOCs) are emitted by the flowers and which are a result of the hydro distillation process. Gas chromatography-olfactometry is one of the possibilities for such an investigation, but at the same time, it is limited by the needed specific equipment and the fact that it remains a subjective descriptive analysis. The headspace sampling, as a nondestructive technique, which can be used on living plants or freshly collected flowers, has been found to be the method of choice in many studies [6,7,8].

Several different headspace techniques (vacuum headspace technique, closed-loop stripping method, dynamic headspace technique, and solid-phase microextraction) [9,10,11] are commonly used to analyze plant VOCs. Among them, solid-phase microextraction (SPME), where various porous polymers are used as fiber materials, has been increasingly applied in the analysis of plant volatiles, including rose aroma. Compared with conventional extraction techniques, this method is convenient, easy to use, less time-consuming, and being solvent-free is truly green. In this way, HS-SPME provides one of the best platforms for sample preparation, especially for the analysis of volatile and semi-volatile organic compounds, which defines its wide practical use [9,10,11,12,13].

The floral scent (as a chemical composition) from two distinct genotypes of Iranian *R. damascena* flowers was analyzed at six stages of flower development by using a simple HS extraction. The main floral components were 2-phenyl ethyl alcohol, *β*-citronellol, *α*-pinene, benzyl alcohol, and geranyl acetate, with variations depending on the stage of development [14].

Three varieties of Damask rose (‘Four Seasons’, ‘Celsiana’, and ‘Rose de Rescht’) were studied in China in a dry and frozen climate [15]. The volatiles emitted from flowers were collected by an improved dynamic HS air-circulation method [16] and were detected and identified via thermal-desorption cold trap-GC/MS technique. The results revealed that 63, 68, and 55 species of individual compounds were detected, respectively.

*R. damascena* Mill. volatiles were studied by HS-SPME at various conditions (fresh, stored at 4 °C, and dried rose petals), identifying 20 volatile compounds [17]. Polydimethylsiloxane (PDMS) coated fused-silica fibers were used as the most suitable ones for adsorbing volatile compounds from the rose petals [18]. The detected compounds varied according to the various storage and drying conditions. It was determined that phenylethyl alcohol, citronellol, geranyl acetate, and nonadecane were predominant compounds in all treatments. Similar analysis was performed by Bydar and co-authors [19] by using carboxen/polydimethylsiloxane fibers, showing that considerable variability in floral scent molecules such as phenylethyl alcohol (23.26–74.54%), citronellol (5.57–31.59%), and geraniol (3.09–26.93%) was recorded among the seed-derived plants.

Rose scent of Egyptian *R. damascena* Mill. unexpanded and expanded rose flowers at different levels of blooming were determined using HS-SPME [20]. SPME fibers coated with divinylbenzene/carboxen/polydimethylsiloxane (DVB/CAR/PDMS, 50/30 µm), PDMS (polydimethylsiloxane) were used. A total of 75 volatiles were detected, with alcohols, esters, and oxides as valuable volatile classes at ca. 64%, 10%, and 2%, respectively, in expanded flowers compared to 46%, 2%, and 0.4% in unexpanded ones.

Joichi and co-workers [21] used dynamic and static HS to investigate the aroma profile of modern tea-hybrid roses ‘Lady Hillingdon’, ‘Diorama’, and ‘Grand Mogul’ along with ancient Chinese roses in order to assess the genealogy of the latter. A substantial number of Seventy-two volatile components from the flowers (‘Lady Hillingdon’—72, ‘Diorama’—76, and ‘Grand Mogul’—67) were identified. A HS-SPME method in combination with gas chromatography (GC) coupled with flame ionization detection (FID) and MS was developed and optimized to investigate volatile organic compounds from different tissues (flowers, leaves, stems, rhizosphere, and whole plants) of Floribunda and Hybrid Tea roses (intact and cut) [22]. Three-phase fiber 50/30 µm divinylbenzene/carboxen/polydimethylsiloxane (DVB/CAR/PDMS) was used. Three taxa of *Rosa odorata* and three taxa of *R. chinensis* germplasm resources were examined for their VOC profiles at different flowering stages by sensory analysis and SPME-GC/MS analysis [23]. DVB/CAR/PDMS was used as an extraction fiber. More than one hundred VOCs were detected, including 33 major compounds. The performed principal component analysis showed characteristic substances such as some benzodiazepines and sesquiterpenoids, which could be used to separate *R. odorata* and *R. chinensis*.

In addition to the rose flowers, the HS-SPME (CAR/PDMS fiber) techniques find applications in the analysis of rose aroma products such as concrete, absolute, and oil from *R. damascena* Mill. [24]. Volatile compounds in the *R. damascena* first rose oil and first rose water have been determined by HS-SPME-GC/MS, showing twenty-four and twenty-six volatile compounds in the former and latter [25].

The technique has promising applications in the analysis of the aroma of medicinal plants [26,27,28], rice [29], and food [13,30,31,32].

Although there are recent studies about the chemical composition of the white oil-bearing rose essential oil [1,2] and its cytotoxic and genotoxic potential [2,3,4,5], there are no published data about the use of HS-SPME for *R. alba* VOCs profiling. The aim of the current study is to fill this research gap. On one side, SPME is one of the ‘greenest’ technologies for sample preparation as it is a solvent-free extraction technology, SPME fibers are reusable, and generate as little waste as possible. On the other hand, it is known that the chemical composition of the rose essential oil does not reflect the true aroma profile of the rose blossom.

Therefore, the first comprehensive VOCs profiling of fresh, unprocessed *R. alba* blossom was performed via HS-SPME using three-phase fibers (DVB/CAR/PDMS) in combination with GC/MS/FID, focusing on the aroma-bearing compounds. The acquired information could have a practical effect in line with the growing interest in *R. alba* as an industrially important alternative to *R. damascena* in perfumery, cosmetics, and pharmaceutical applications.

## 2. Results and Discussion

The analysis was performed by means of HS-SPME-GC/MS, which allows efficient extraction and concentrates aromatic compounds. In this study, a semi-quantitative HS-SPME-GC/MS/FID method has been developed for a fresh *R. alba* blossom aroma profile analysis. Both GC/MS and GC-FID are powerful and sensitive techniques for analysis of volatile and semi-volatile compounds in complex mixtures, but GC-FID is more preferable for the quantitation, while GC/MS is unsurpassed for qualitative analysis and identification of the constituents. Therefore, the quantitative results, in relative percents, given in the text below are from the GC-FID analysis (Table 1 and Table S1).

**Figure 1:**
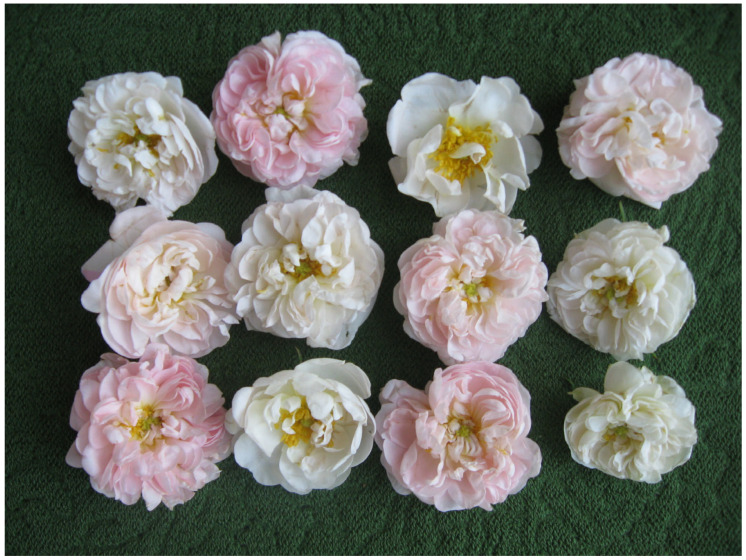
Variations of color tint and number of petals in the *R. alba* population (authors’ picture).

Flowers from two clones of the *R. alba* population were used, with predominantly white and rose-colored petals (Figure 1), labelled as white and pink in the manuscript. Among all of the variations in the population, these two clones are the most promising according to the olfactory estimation and the essential oil composition.

More than 75 individual compounds were detected, and 47 of them were identified and quantified in the fresh *R. alba* L. flowers using HS-SPME-GC/MS/FID.

**Figure 2:**
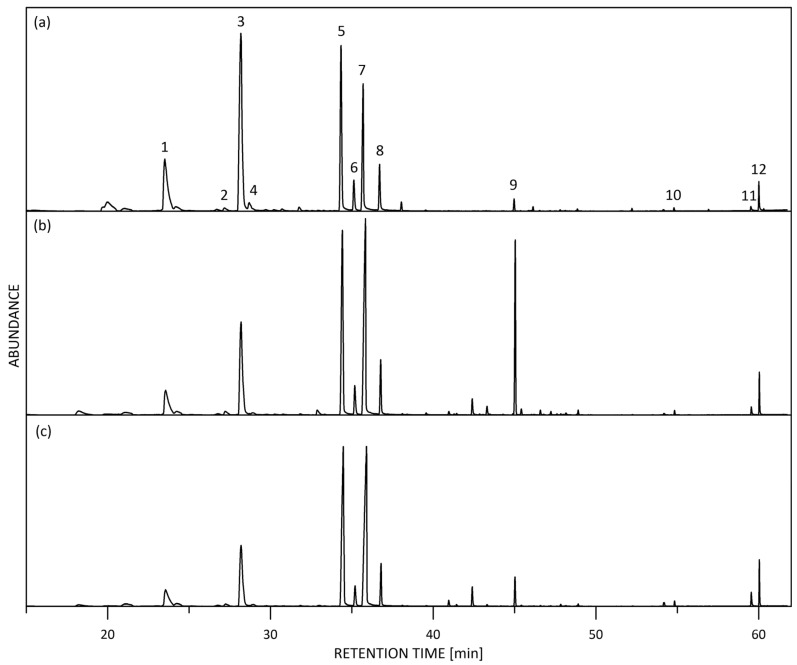
Representative HS-SPME-GC/MS total-ion current (TIC) chromatogram of fresh *R. alba* flowers: (**a**) Petals mix; (**b**) Whole blossom white; (**c**) Whole blossom pink. Main components: 1. Benzyl alcohol, 2. *cis*-Rose oxide, 3. 2-Phenyl ethyl alcohol, 4. *trans*-Rose oxide, 5. *β*-Citronellol + Nerol, 6. *Z*-Citral (Neral), 7. Geraniol, 8. *E*-Citral (Geranial), 9. *trans*-*β*-Caryophyllene, 10. Heptadecane, 11. Nonadecene, 12. Nonadecane.

Representative GC/MS total-ion current (TIC) chromatograms of fresh *R. alba* blossoms are shown in Figure 2.

It is known that the most floral scent compounds are produced via three general biosynthetic pathways, i.e., terpenoids, phenylpropanoids/benzenoids, and fatty-acid derivatives [33]. The oil-bearing rose species are rich in terpenoids and phenylpropanoid/benzenoid compounds, which not only enhance the ornamental value of roses, but are also an industrially important source of fragrances and flavorings for perfumery and cosmetics, and are responsible for the wide range of pharmacological activities.

Our study revealed that the aroma fraction of *R. alba* volatile organic compounds (VOCs) consists mainly of monoterpene alcohols; 2-phenylethanol (8.4–33.9%) was the most abundant component, followed by geraniol (12.8–32.5%) and *β*-citronellol + nerol (17.7–26.5%). Benzyl alcohol and benzaldehyde were found in a relatively broad concentration range (2.62–13.78/0.42–4.04%, respectively). Linalool, *α*-pinene, *β*-myrcene, rose oxides, esters, as well as sesquiterpene compounds were observed in low concentrations. The stearopten fraction in the HS phase was observed at low concentration, with main representatives nonadecane/nonadecene (0.56–10.95/0.21–3.23%), heptadecane, heneicosane, and tricosane (total n-alkane fraction in the range 1.19–17.32%).

Similar to our results, the essential oil from *R. alba* was found to be rich in geraniol (18.28%), followed by heneicosane (12.95%), nonadecane (10.75%), and citronellol (9.00%) [34].

The main components responsible for the aroma profile of the white oil-bearing rose are presented in Table 1, and the quantitative results for all identified VOCs in *R. alba* blossom, together with the aroma description of each compound, are collected in Table S1. In addition, information about the Odor detection (OD) thresholds and CAS numbers of the compounds is presented in the Supplementary Information (Table S2).

**Figure 3:**
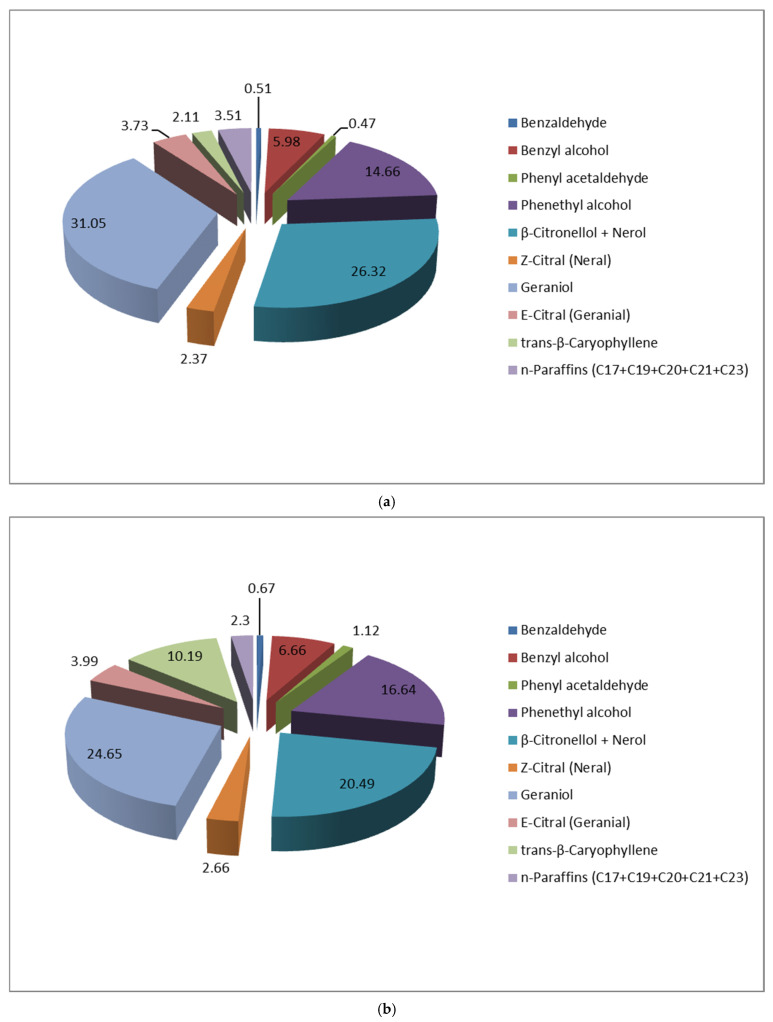
VOCs profile (at 45 °C) of fresh *R. alba* blossom with pink (**a**) and white (**b**) colored petals.

Our study reveals that the two studied *R. alba* population clones with pink and white colored petals show different VOCs profiles (Figure 3).

This finding is in agreement with the previous reported GC-MS analysis of the volatiles of essential oils distilled from two different *R. alba* accessions, which showed significant variation of the abundances of some of the volatiles between the two studied phenogroups [3].

As seen from Figure 1 and Table 1 and Table S1, petals and whole *R. alba* blossoms also show different VOC composition, which agrees with the observation of Ibrahim et al. [22] for different volatiles emitted from different rose tissues (buds, stems, leaves, and flowers).

**Figure 4:**
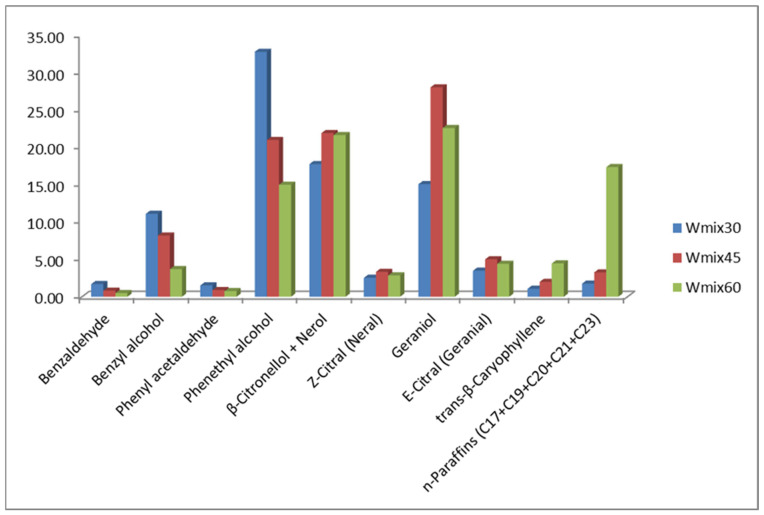
Effect of the extraction temperature (30–60 °C) on the VOCs of *R. alba* mixed whole blossom sample. Legend: Wmix—mixed whole blossom; the numbers 30, 45, 60 indicate SPME performed at 30, 45, 60 °C, respectively.

The effect of different extraction temperatures (30–60 °C) on the *R. alba* volatiles content using whole mixed blossom samples is shown in Figure 4.

In general, increasing the conditioning and extraction temperature reduces the concentration of low-boiling compounds, which is most noticeable by benzyl- and 2-phenyl ethyl alcohol, together with benzaldehyde, phenyl acetaldehyde, and monoterpenes, while the higher temperatures used in SPME experiments increase the amount of high-boiling sesquiterpenes and n-paraffins.

### 2.1. Phenylethanoids and Benzenoids

The characteristic rose-like odor (very lasting, though mild and warm rose and rose-honey note) of Rosaceae plants is due to the phenylethanoid 2-phenylethyl alcohol. However, being highly soluble in water, the 2-phenylethyl alcohol is usually lost in the distillation process (or collected in the rose water), and its concentration in the rose oil, derived by the traditional hydro distillation methods, is low. Our study reveals that 2-phenylethyl alcohol is the most abundant single component in SPME *R. alba* aroma profile, with a maximal concentration of 33.93% in the mixed petals sample, extracted at room temperature. The same sample shows maximal concentration for benzyl alcohol and benzaldehyde, 13.78 and 4.04%, respectively. The higher extraction temperature decreases their amounts, reaching the minimum value of 8.41% for 2-phenylethyl alcohol and 2.62% for benzyl alcohol at 60 °C. In addition, *phenyl acetaldehyde* was found in a maximal concentration of 1.69% in the mixed petals sample.

It is worth mentioning that methyl eugenol, a phenyl propanoid, which is naturally occurring in *R. damascena* as a minor component and its content is regulated by the ISO 9842:2024 [35] due to its allergenic and potentially cancerogenic properties, is absent in *R. alba* fresh blossom VOCs. This fact is in agreement with the studies about the chemical composition of the *R. alba* essential oil [1], making white oil-bearing rose a prospective alternative to Damask rose for perfumery, cosmetics, and medical applications, with a very low toxic potential [2,4,5].

### 2.2. Terpenoids

#### 2.2.1. Monoterpenes

The monoterpene hydrocarbons total content is in a relatively narrow concentration range from 1.65 to 4.19%, with the main representative unsaturated bicyclic monoterpene pinene (α- and β-). Pinene isomers add pine- and turpentine-like notes to the fragrance profile. It is important to underline that *α*-pinene was observed in whole *R. alba* blossoms only and was not detected in rose petals, with a maximal concentration of 1.62% in white colored samples. Its isomer *β*-pinene was found in a maximal concentration of 2.01% in samples with pink colored petals. The high extraction temperature reduces the content of both pinene isomers—the lowest concentration (0.42% for *α*- and 0.72% for *β*-pinene) was observed by extraction at 60 °C, while the SPME at room temperature increases pinene content. The acyclic monoterpene β-myrcene was found in trace amounts only.

Oxygenated monoterpenes

Monoterpene alcohols. The most abundant class of aroma compounds presented in the *R. alba* VOCs profile is the monoterpene alcohols. Geraniol, nerol, and citronellol are the main compounds responsible for the fragrance and biological activity of the oil-bearing rose species and their aroma products (and are often being used for adulteration of the high-priced rose aroma products with synthetic compounds). Citronellol and nerol give the characteristic rosaceous essence. Geraniol is also basic for the scent and exerts the major pharmacological effects of the rose oil, such as antitumor, antibacterial, antifungal, antioxidant, and anti-inflammatory [36]. In this study, geraniol was the second most abundant single component, followed by citronellol and nerol. In general, their amounts in rose petals were found to be higher in comparison to the whole *R. alba* blossom—geraniol shows a maximal concentration of 32.45% in white petals sample, while citronellol/nerol were observed in maximal concentration in mixed petals sample (26.5%). Linalool, known as one of the rose alcohols, adding rich floral and spicy notes to the rose aroma, was found in a maximal concentration of 0.81% in a whole blossom sample extracted at 30 °C.

Ketones. It is generally accepted that rose ketones are very important for rose fragrance. *β*-damascone (1-(2,6,6-trimethyl-1-cyclohexenyl)but-2-en-1-one), together with *β*-damascenone (1-(2,6,6-trimethyl-1-cyclohexa-1,3-dienyl)but-2-en-1-one), plays an important organoleptic role as a trace component and as a quality marker of Bulgarian rose oil. In our study, *β*-damascone was found in a very low concentration <LOQ (0.005%) in fresh *R. alba* flowers, while *β*-damascenone was not detected.

Oxides. Tetrahydro-4-methyl-2-(2-methylpropenyl)-2H-pyran (rose oxide), existing as *cis*- and *trans*-isomers, is a key minor component of the rose essential oil. In the current study, *cis*-rose oxide was observed in 0.03–0.12%, while the *trans*-isomer was found in a higher concentration (0.08–0.29%).

These results are very important, because it has been previously reported and generally accepted that the isomeric rose oxides do not occur in the rose flowers, because these volatiles appear to be artefacts formed during the steam distillation process [6]. Our study undoubtedly confirms the presence of the rose ketones in fresh *R. alba* flowers.

#### 2.2.2. Sesquiterpenoids

Sesquiterpenoids were found mainly in non-oxygenated form, with *trans*-*β*-carryophylene as the main representative. It was found in a maximum amount of 10.19% in whole blossom with white colored petals, while the pink colored whole blossom sample shows the minimum concentration (0.25%). Other sesquiterpene hydrocarbons contributing to the *R. alba* VOCs profile as minor components were germacrene-D, *α*-humulene, *α*-caryophyllene, *γ*-murolene, etc. The sesquiterpene alcohol *trans*, *trans*-*β*-farnesol was found in trace amounts only.

### 2.3. Esters

Esters of 2-phenylethyl alcohol and the most abundant monoterpene alcohols geraniol, citronellol, and nerol, with acetic acid, were mainly found in a total amount of 0.15–4.79%.

### 2.4. Stearopten

Aliphatic hydrocarbons (alkanes and alkenes), as a part of the rose plant wax and forming the solid part of distilled rose oil (so-called stearopten), are odorless, but they play a significant role in the aroma products as compounds, responsible for the odor stability. In the HS-SPME profile of *R. alba* blossom, they were presented with main representatives nonadecane/nonadecene (0.56–10.95/0.21–3.23%), heptadecane, heneicosane, and tricosane. The total amount of the n-paraffin fraction was in the range 1.19–17.32%.

### 2.5. Key Aroma Compounds in R. alba Fresh Blossom

Usually, the characteristic rose-like scent is correlated with the high amounts of *β*-citronellol, nerol, geraniol, and 2-phenylethyl alcohol. Our study reveals that these four components are also key aroma compounds in the VOC profile of fresh *R. alba* blossom, together with phenyl acetaldehyde, linalool, geranyl acetate, and *E*- and *Z*-citral. Based on the OD thresholds approach [37], some components, even presenting in low concentrations, could have a huge impact on the fragrance emitted by rose flowers. It is very important to note that the rose oxides and *β*-damascone, which have been found in the fresh *R. alba* blossom as minor components, with their very low OD thresholds—0.5 ppb for *cis*-rose oxide and 1.5 ppb for *β*-damascone (Table S2, Supplementary Information), are extremely important key aroma compounds in the rose aroma profile.

## 3. Materials and Methods

### 3.1. Materials

The experiments were conducted in May 2024. Fresh rose blossoms of *Rosa alba* L. picked up early in the morning (6–8 a.m.), from a 10-year-old plantation of the Experimental field in the Institute of Roses and Aromatic Plants, Kazanlak, Bulgaria, were used as raw material. The rose blossom was picked in the most suitable development stage, semi- to fully-open, when the flowers emit the most diversified aroma compounds, reaching the maximum of the relative abundance. The population consists of four clones, which differ mainly in the number of petals and the color tint [1]. In the current study, flowers from two clones were used, with predominantly white and rose-colored petals (Figure 1), labelled as white and pink in the manuscript, picked and studied separately, as well as a mix of them. In addition, samples from whole *R. alba* blossoms and only petals were also analyzed separately. Every sample consists of 2.5 ± 0.25 g plant material, weighed immediately after collecting the plant material, sealed in 25 mL headspace vials, and stored in a refrigerator at −18 °C before the GC/MS analysis.

N-Alkane mixture C7-C30 (TraceCERT^®^ certified reference material, 1000 μg/mL each component in hexane, Supelco, Bellefonte, PA, USA) was used for the retention indices determination.

Divinylbenzene/carboxen/polydimethylsiloxane (DVB/CAR/PDMS) 50/30 µm SPME Fiber Assembly ((50 μm DVB layer, 30 μm CAR/PDMS layer), Supelco, Bellefonte, PA, USA) for use with an autosampler was used.

### 3.2. Methods

#### 3.2.1. SPME

SPME uses a coated fiber to concentrate volatile and semi-volatile compounds from a sample. In this extraction technique, analytes establish equilibria among the sample matrix, the headspace above the sample, and the polymer-coated fused phase. There are two most commonly used modes of SPME extractions, HS (most common) and direct immersion (DI), where the SPME fiber is immersed in the aqueous sample (specific applications). In our study, the HS-SPME mode was used.

The SPME experiments were performed on an HT2800T all-in-one GC autosampler (HTA s.r.l., Brescia, Italy), working in liquid, static HS, and SPME modes. Mid-polar three-phase DVB/CAR/PDMS 50/30 µm fibers were used for extracting volatiles. This three-phase fiber coating is especially suitable for analyte group flavors (volatiles and semi-volatiles, with carbon numbers C3-20, MW in the range 40–275). An optimization of the experimental conditions (conditioning temperature (30–60 °C), conditioning and extraction time (15–45 min)) was performed to study their influence on the qualitative and quantitative content of the VOCs extracted from the rose samples. The conditions used for the optimization of the SPME experiments are collected in Table S3 (Supplementary Information). The parameters used for the optimization were chosen in a very practical way on the basis of equipment limitations and optimal sample run duration. The lowest temperature of the HT2800T oven is 30 °C, and the 15 °C step is reasonable to test the effect of the extraction temperature. The optimal duration of the conditioning and extraction was 30 min, chosen on the basis of the number and abundance of the detected VOCs, and all the experiments were performed under these conditions, in three replicates.

#### 3.2.2. Gas Chromatography-Mass Spectrometry (GC/MS)

The GC/MS analysis was performed on an Agilent 7820A GC System Plus gas chromatograph coupled with a 5977 B Mass Selective detector and flame-ionization detector (Agilent Technologies, Palo Alto, CA, USA). A fused silica capillary column, a mid-polar DB-17HT (J&W Scientific, Folsom, CA, USA) with 60 m column length, 0.25 mm i.d., 0.25 μm film thickness, was used. The oven temperature was programmed from 60 °C (2.5 min held) to 100 °C at a rate of 5 °C/min, from 100 to 225 °C at a rate of 2.5 °C/min, and from 225 to 275 °C at a rate of 5 °C/min. A 10-min hold at the final temperature was applied. Helium (99.999%) was used as a carrier gas at a constant flow rate of 0.8 mL/min. The split ratio was 1:125, the inlet temperature was set to 260 °C, and the transfer line temperature was 280 °C. Mass selective detector operated in electron impact ionization (EI) mode at 70 eV electron energy, the ion source temperature was set to 230 °C, and the quadrupole temperature was 150 °C. The mass scan range was 45–1050 m/z.

#### 3.2.3. Gas Chromatography with Flame-Ionization Detector (GC-FID)

The GC-FID analysis was performed on the same instrument under the same temperature gradient as described above. The system is equipped with a post-column split of the flow, allowing simultaneous analysis on both detectors. Instrument control and data collection were carried out using Mass Hunter Workstation Software (Revision B.06.07, Agilent Technologies, Santa Clara, USA).

#### 3.2.4. Identification and Quantitative Analysis

The identification of the compounds was performed by GC/MS, using commercial mass spectral libraries (NIST 14, Wiley 7th Mass spectra register, and FFNSC3 Flavor and Fragrance GC/MS library) and retention times (Linear retention indices, LRI). In the cases of a lack of corresponding reference data, the structures were proposed based on their general fragmentation pattern and/or using the reference literature mass spectra. The quantification of the main compounds was carried out by GC-FID using an Internal normalization method with the response factor set equal to unity for all of the sample constituents. Although not being considered as a true quantification, a simple GC-FID percentage allows for a comparison between the rose samples studied. In addition, this procedure is used in the ISO 9842:2024 [35] and provides results that are comparable with most of the data for rose oils reported in the literature.

## 4. Conclusions

The first comprehensive VOCs profiling of fresh, unprocessed *R. alba* blossom was performed using the developed HS-SPME-GC/MS/FID method with three-phase coated fibers (DVB/CAR/PDMS).

The studied *R. alba* population clones with pink and white colored petals show different VOC profiles. A distinct difference between petals and whole blossom VOC compositions is observed as well.

In the current study, rose oxides, key aroma compounds of rose essential oil, were detected in the fresh *R. alba* blossom. This is a very important result, because it has been generally accepted that the isomeric rose oxides do not occur in the rose flowers, because these volatiles appear to be artefacts formed during the steam distillation process.

It is also worth mentioning the absence of some undesirable components, such as allergenic and potentially carcinogenic methyl eugenol in fresh *R. alba* blossom, which makes *R. alba* a promising alternative to *R. damascena* in perfumery, cosmetics, aromatherapy, and pharmaceutical applications.

## Figures and Tables

**Table 1:** Main aroma-bearing components, observed in *R. alba* L. fresh blossom by HS-SPME-GC. Legend: W-whole blossom, P-petals, the numbers 30, 45, 60 indicate the SPME performed at 30, 45, 60 °C, respectively.

No	Compound	Aroma Description	*R. alba* HS-SPME VOCs, in Relative % (Averaged from 3 Replicates), as Measured by GC/FID														Min	Max
			Wmix30	Wmix45	Wmix60	Pmix 30	Pmix 45	Pmix 60	Wpink30	Wpink45	Wwhite30	Wwhite45	Ppink30	Ppink45	Pwhite30	Pwhite45		
1	*α*-Pinene	Herbal type	1.18	0.93	0.42	n.d	n.d	n.d	1.49	1.01	1.12	1.64	n.d	n.d	n.d	n.d	0.42	1.64
2	Benzaldehyde	Sharp sweet bitter-almond cherry	1.69	0.81	0.48	4.04	0.69	0.42	1.64	0.51	1.02	0.67	0.49	0.61	0.47	0.75	0.42	4.04
3	*β*-Pinene/*β*-Myrcene	Woody-green pine like/Sweet-balsamic-resinous	1.51	0.98	0.72	1.49	0.97	0.92	1.23	2.01	0.95	1.27	1.65	1.18	1.16	1.27	0.72	2.01
4	Benzyl alcohol	Slightly sweet, floral	11.08	8.18	3.71	13.78	7.95	2.62	12.73	5.98	10.15	6.66	9.84	7.54	7.37	8.49	2.62	13.8
5	Phenyl acetaldehyde	Green, floral, reminiscent of Lilac	1.51	0.88	0.74	1.30	0.85	0.79	0.85	0.47	0.95	1.12	0.73	0.95	0.77	1.19	0.47	1.51
6	Linalool	Floral, spicy wood	0.81	0.54	0.48	0.53	0.56	0.49	0.64	0.65	0.5	0.77	0.52	0.61	0.39	0.65	0.39	0.81
7	*cis*-Rose oxide	Typical rose, floral-green	0.05	0.03	0.11	0.02	0.03	0.09	0.02	0.04	0.02	0.05	0.12	0.03	0.06	0.02	0.02	0.12
8	2-Phenyl ethyl alcohol	Rose note, very lasting/mild and warm rose honey	32.74	20.96	14.97	33.93	21.64	8.41	20.45	14.66	31.67	16.64	26.35	19.07	21.91	23.29	8.41	33.9
9	*trans*-Rose oxide	Floral-green, herbal (minty) and fruity	0.37	0.05	0.46	0.45	0.26	0.43	0.54	0.08	0.2	0.1	0.14	0.59	0.09	0.15	0.05	0.59
10	*β*-Citronellol + Nerol	Sweet, rose like/Rose like, fresh green note	17.72	21.88	21.6	18.19	21.83	26.54	20.05	26.32	21.78	20.49	24.74	25.9	24.74	21.1	17.7	26.5
11	*Z*-Citral (Neral)	Citrus, milder, and sweeter	2.53	3.33	2.84	2.41	2.52	3.23	3.15	2.37	3.5	2.66	1.23	2.19	4.88	1.77	1.23	4.88
12	Geraniol	Sweet, floral, rose-like	15.04	27.99	22.56	12.75	31.59	29.12	19.84	31.05	18.32	24.65	20.1	31.9	24.62	32.45	12.8	32.5
13	*E*-Citral (Geranial)	Strong, lemon like	3.48	4.99	4.4	3.18	3.75	4.99	5.31	3.73	5.12	3.99	1.49	3.53	6.93	2.7	1.49	6.93
14	Geranyl acetate	Floral, fruity, rose like	0.09	0.37	0.45	0.21	0.36	0.97	0.66	1.54	0.46	1.09	3.51	0.77	2.48	0.78	0.09	3.51
15	*β*-Damascone	Intense rose-like	0.21	0.11	0.09	0.27	0.10	0.1	0.09	0.09	0.03	0.08	0.05	0.09	0.19	0.10	0.03	0.27
16	*trans*-*β*-Caryophyllene	Softly spicy, woody	1.06	1.99	4.44	0.67	0.27	0.46	1.01	2.11	1.19	10.19	0.13	0.24	0.19	0.11	0.11	10.2
17	Pentadecane (C15)	Odorless	0.09	0.11	0.18	0.09	0.14	0.39	0.1	0.14	0.06	0.06	0.12	0.09	0.1	0.08	0.06	0.39
18	Heptadecene (C17:1)	Odorless	0.17	0.25	0.75	0.14	0.39	1.35	0.22	0.36	0.17	0.11	0.17	0.33	0.18	0.19	0.11	1.35
19	Heptadecane (C17)	Odorless	0.28	0.29	1.4	0.19	0.41	1.83	0.18	0.32	0.21	0.22	0.21	0.28	0.18	0.23	0.18	1.83
20	Nonadecene (C19:1)	Odorless	0.37	0.71	3.2	0.23	1.09	3.21	0.45	0.91	0.4	0.43	0.63	0.84	0.45	0.68	0.23	3.21
21	Nonadecane (C19)	Odorless	1.11	2.16	10.95	0.91	2.19	8.72	0.56	2.15	0.82	1.63	2.06	1.91	0.91	1.61	0.56	11.0
TOTAL																		
Monoterpene Hydrocarbons			4.19	2.79	1.88	2.78	1.82	1.71	2.72	3.49	2.07	4.03	1.65	2.13	1.93	2.46	1.65	4.03
Monoterpene Oxidized			40.8	59.3	52.5	38.0	60.9	65.0	49.7	64.7	50.1	53.0	48.6	64.7	62.1	59.2	48.6	65.0
Sesquiterpene Hydrocarbons			1.5	2.55	5.79	1.09	0.68	1.20	1.25	2.49	1.50	10.96	0.25	0.42	0.50	0.42	0.25	11.0
Esters			0.36	0.84	1.01	0.15	0.79	1.71	0.99	2.26	0.85	1.73	4.79	1.20	3.09	1.14	0.85	4.79
n-Paraffins (C17 + C19 + C20 + C21 + C23)			1.76	3.25	17.32	1.33	3.88	14.88	1.19	3.51	1.43	2.30	3.17	3.18	1.66	2.63	1.19	14.9

## Data Availability

The original contributions presented in this study are included in the article/Supplementary Material. Further inquiries can be directed to the corresponding authors.

## Supplementary Materials

The following supporting information can be downloaded at https://www.mdpi.com/article/10.3390/molecules30153102/s1: Table S1: VOCs profile, observed in *R. alba* L. fresh blossom by HS-SPME-GC, Mean (in rel. %) ± SD; Table S2: Main components, observed in *R. alba* L. fresh blossom by HS-SPME-GC, with CAS-numbers and OD thresholds; Table S3. SPME experimental conditions used for the method optimization.

## Abbreviations

The following abbreviations are used in this manuscript:

GC/MS	Gas chromatography-Mass spectrometry
GC-FID	Gas chromatography with flame ionization detection
HS	Headspace
SPME	Solid-phase microextraction
TIC	Total-ion current
VOCs	Volatile organic compounds
DVB/CAR/PDMS	Divinylbenzene/Carboxen/Polydimethylsiloxane
LOQ	Limit of quantitation
SD	Standard deviation
RI	Retention Index
n.d.	not detected
W	whole blossom
W_mix_n (n = 30, 45, 60 °C (SPME temperature))	Whole blossom, mixed sample
W_white_n (n = 30, 45, 60 °C (SPME temperature))	Whole blossom, white
W_pink_n (n = 30, 45, 60 °C (SPME temperature))	Whole blossom, pink
P	petals
P_mix_n (n = 30, 45, 60 °C (SPME temperature))	Petals, mixed sample
P_pink_n (n = 30, 45, 60 °C (SPME temperature))	Petals, pink
P_white_n (n = 30, 45, 60 °C (SPME temperature))	Petals, white-coloured sample
