# Supplementary material for: Volatile Compounds Profiling of Fresh R. alba L. Blossom by Headspace—Solid Phase Microextraction and Gas Chromatography
Source: Molecules. 2025 Jul 24;30(15):3102. doi: 10.3390/molecules30153102 (PMC12348832; doi:10.3390/molecules30153102)
Supplement: Supplementary file 1 [file molecules-30-03102-s001.zip › molecules-3733542-supplementary.pdf]

Supplementary Information

No	Compound	Aroma description	RI exp	RI lit	HS-SPME VOCs profile of *R.alba* blossom, in relative %, as measured by GC/FID													
					$W_{mix}30$	$W_{mix}45$	$W_{mix}60$	$P_{mix}30$	$P_{mix}45$	$P_{mix}60$	$W_{pink}30$	$W_{pink}45$	$W_{white}30$	$W_{white}45$	$P_{pink}30$	$P_{pink}45$	$P_{white}30$	$P_{white}45$
1	**α-Pinene**	Herbal type	932	939	1.18 ±0.01	0.93 ±0.07	0.42 ±0.04	n.d	n.d	n.d	1.49 ±0.09	1.01 ±0.06	1.12 ±0.05	1.64 ±0.28	n.d	n.d	n.d	n.d
2	**Benzaldehyde**	Sharp sweet bitter-almond cherry	962	969	1.69 ±0.24	0.81 ±0.15	0.48 ±0.09	4.04 ±0.51	0.69 ±0.08	0.42 ±0.04	1.64 ±0.18	0.51 ±0.04	1.02 ±0.05	0.67 ±0.03	0.49 ±0.02	0.61 ±0.06	0.47 ±0.05	0.75 ±0.07
3	**β-Pinene/β-Myrcene**	Woody-green pinelike/Swe t-balsamic-resinous	988	981	1.51 ±0.04	0.98 ±0.02	0.7 2±0.03	1.49 ±0.03	0.97 ±0.02	0.92 ±0.02	1.23 ±0.03	2.01 ±0.04	0.95 ±0.05	1.27 ±0.03	1.65 ±0.06	1.18 ±0.06	1.16 ±0.06	1.27 ±0.06
4	**Benzyl alcohol**	Slightly sweet, floral	1036	1035	11.08 ±0.14	8.18 ±0.13	3.71 ±0.07	13.78 ±0.2	7.95 ±0.18	2.62 ±0.06	12.73 ±0.18	5.98 ±0.08	10.15 ±0.16	6.66 ±0.09	9.84 ±0.18	7.54 ±0.48	7.37 ±0.32	8.49 ±0.22
5	**Phenyl acetaldehyde**	Green, floral, reminiscent of Lilac	1045	1045	1.51 ±0.06	0.88 ±0.08	0.74 ±0.05	1.30 ±0.06	0.85 ±0.03	0.79 ±0.02	0.85 ±0.03	0.47 ±0.02	0.95 ±0.07	1.12 ±0.03	0.73 ±0.04	0.95 ±0.09	0.77 ±0.08	1.19 ±0.09
6	**α-Terpinolene+Rose furan**	Fresh, woody, pine, turpentine- like	1081	1083	0.34 ±0.03	0.12 ±0.02	0.15 ±0.02	0.44 ±0.03	0.21 ±0.02	0.21 ±0.02	0.64 ±0.04	0.33 ±0.02	0.20 ±0.02	0.30 ±0.02	1.49 ±0.06	0.21 ±0.04	0.49 ±0.04	0.26 ±0.03
7	**Linalool**	Floral, spicy wood	1100	1099	0.81 ±0.12	0.54 ±0.06	0.48 ±0.04	0.53 ±0.05	0.56 ±0.04	0.49 ±0.04	0.64 ±0.05	0.65 ±0.04	0.5±0.03	0.77 ±0.04	0.52 ±0.04	0.61 ±0.05	0.39 ±0.03	0.65 ±0.05
8	***cis*-Rose oxide**	Typical rose, floral-green	1111	1111	0.05 ±0.01	0.03 ±0.01	0.11 ±0.03	0.02 ±0.01	0.03 ±0.01	0.09 ±0.02	0.02 ±.01	0.04 ±0.01	0.02 ±0.01	0.05 ±0.01	0.12 ±0.02	0.03 ±0.01	0.06 ±0.01	0.02 ±0.01
9	**Phenethyl alcohol**	Rose note, very lasting/mild and warm rose honey	1115	1117	32.74 ±3.39	20.96 ±2.24	14.97 ±1.48	33.93 ±0.6	21.64 ±1.7	8.41 ±0.74	20.45 ±2.31	14.66 ±0.98	31.67 ±1.24	16.64 ±1.12	26.35 ±0.78	19.07 ±1.03	21.91 ±1.05	23.29 ±0.75
10	***trans*- Rose oxide**	Floral-green, herbal (minty) and fruity	1128	1126	0.37 ±0.02	0.05 ±0.01	0.46 ±0.08	0.45 ±0.03	0.26 ±0.02	0.43 ±0.02	0.54 ±0.04	0.08 ±0.02	0.20 ±0.03	0.10 ±0.02	0.14 ±0.03	0.59 ±0.03	0.09 ±0.02	0.15 ±0.03
11	**Citronellal (6-Octenal, 3,7-dimethyl-)**	Citrus, lemongrass	1149	1153	0.17 ±0.01	0.11 ±0.01	0.09 ±0.02	0.09 ±0.01	0.10 ±0.01	<LOQ	0.24 ±0.02	0.08 ±0.01	0.40 ±0.02	0.12 ±0.02	0.13 ±0.03	0.10 ±0.02	0.14 ±0.02	<LOQ
12	***i*-Neral**	Green	1264	1165	0.35 ±0.04	0.15 ±0.03	0.13 ±0.02	0.23 ±0.01	0.13 ±0.02	0.16 ±0.03	0.21 ±0.02	0.07 ±0.02	0.20 ±0.02	0.09 ±0.02	0.23 ±0.04	0.11 ±0.02	0.08 ±0.02	0.12 ±0.02
14	**Terpinen-4-ol**	Mild earthy and woody	1186	1179	0.09 ±0.02	<LOQ	<LOQ	<LOQ	<LOQ	<LOQ	<LOQ	<LOQ	<LOQ	<LOQ	<LOQ	<LOQ	0.21 ±0.04	<LOQ
15	***i*-Geranial**	Green	1181	1184	0.51 ±0.06	0.27 ±0.03	0.23 ±0.03	0.43 ±0.05	0.19 ±0.03	0.25 ±0.03	0.22 ±0.02	0.13 ±0.02	0.28 ±0.03	0.14 ±0.03	0.06 ±0.02	0.17 ±0.01	0.12 ±0.02	0.17 ±0.03
16	**α-Terpineol**	Floral, sweet, lilac-type	1200	1197	0.08 ±0.02	0.07 ±0.01	0.15 ±0.02	0.09 ±0.02	0.19 ±0.02	0.19 ±0.02	0.07 ±0.01	0.21 ±0.02	0.07 ±0.01	0.11 ±0.02	0.09 ±0.02	0.19 ±0.01	0.07 ±0.01	0.21 ±0.06
17	***cis*-Carveol**	Spearmint-like	1221	1229	0.07 ±0.00	0.08 ±0.01	0.06 ±0.01	0.08 ±0.02	0.07 ±0.01	0.06 ±0.01	0.05 ±0.01	0.07 ±0.01	0.05 ±0.01	0.08 ±0.01	0.07 ±0.01	0.07 ±0.00	0.06 ±0.01	0.07 ±0.01
18	**β-Citronellol + Nerol**	Sweet, rose like/Rose like, fresh green note	1228	1223	17.72 ±1.24	21.88 ±0.98	21.6 ±0.76	18.19 ±1.1	21.83 ±1.1	26.54 ±1.7	20.05 ±1.17	26.32 ±1.87	21.78 ±0.54	20.49 ±0.67	24.74 ±0.98	25.90 ±0.55	24.74 ±0.89	21.1 ±0.46
19	***Z*-Citral (Neral)**	Citrus, milder, and sweeter	1242	1247	2.53 ±0.34	3.33 ±0.27	2.84 ±0.16	2.41 ±0.27	2.52 ±0.15	3.23 ±0.09	3.15 ±0.08	2.37 ±0.07	3.5 ±0.11	2.66 ±0.06	1.23 ±0.03	2.19 ±0.19	4.88 ±0.21	1.77 ±0.07
20	**Geraniol**	Sweet,floral, rose-like	1254	1249	15.04 ±2.15	27.99 ±2.32	22.56 ±1.97	12.75 ±1.9	31.59 ±2.5	29.12 ±1.9	19.84 ±1.13	31.05 ±2.45	18.32 ±1.08	24.65 ±2.11	20.1 ±1.81	31.9 ±1.28	24.62 ±1.23	32.45 ±0.98
21	**Phenyl ethyl acetate**	Sweet, rosy-fruity, honey-like	1256	1258	<LOQ	<LOQ	<LOQ	<LOQ	<LOQ	<LOQ	<LOQ	<LOQ	<LOQ	<LOQ	<LOQ	<LOQ	<LOQ	<LOQ
22	***E*-Citral (Geranial)**	Strong, lemon like	1270	1277	3.48 ±0.24	4.99 ±0.34	4.40 ±0.11	3.18 ±0.18	3.75 ±0.21	4.99 ±0.24	5.31 ±0.21	3.73 ±0.09	5.12 ±0.08	3.99 ±0.09	1.49 ±0.03	3.53 ±0.07	6.93 ±0.09	2.7 ±0.07
23	**Methyl geranate**	Mainly floral and	1322	1321	0.11	0.19	0.13	0.11	0.23	0.26	0.19	0.07	0.09	0.16	0.25	0.06	0.08	0.07

		herbal aroma, but			±0.01	±0.02	±0.02	±0.02	±0.03	±0.03	±0.02	±0.02	±0.02	±0.03	±0.02	±0.01	±0.02	±0.01
24	**Citronellyl acetate**	Fresh-rosy, fruity	1350	1357	0.12 ±0.01	0.11 ±0.01	0.14 ±0.02	0.11 ±0.01	0.11 ±0.02	0.26 ±0.02	0.12 ±0.02	0.48 ±0.03	0.20 ±0.02	0.24 ±0.02	0.45 ±0.02	0.23 ±0.01	0.32 ±0.02	0.14 ±0.04
25	***α*-Cubebene**	Warm woody, slightly camphoraceous	1352	1351	0.02 ±0.01	0.03 ±0.01	0.02 ±0.01	<LOQ	<LOQ	<LOQ	<LOQ	0.02 ±0.01	0.03 ±0.01	0.06 ±0.01	0.11 ±0.03	0.03 ±0.01	0.26 ±0.02	0.12 ±0.03
26	**Neryl acetate**	Fresh, fruity floral, rose and geranium aspects	1359	1362	0.06 ±0.01	0.06 ±0.01	0.05 ±0.02	0.05 ±0.01	0.06 ±0.01	0.14 ±0.02	0.10 ±0.03	0.15 ±0.02	0.05 ±0.01	0.09 ±0.02	0.51 ±0.04	0.09 ±0.02	0.19 ±0.03	0.10 ±0.09
27	**Geranyl acetate**	Floral, fruity, rose like	1378	1379	0.09 ±0.02	0.37 ±0.05	0.45 ±0.05	0.21 ±0.04	0.36 ±0.06	0.97 ±0.07	0.66 ±0.06	1.54 ±0.05	0.46 ±0.04	1.09 ±0.09	3.51 ±0.07	0.77 ±0.08	2.48 ±0.06	0.78 ±0.03
28	***β*-Damascone**	Intense rose-like	1380	1385	0.21 ±0.04	0.11 ±0.03	0.09 ±0.02	0.27 ±0.04	0.10 ±0.02	0.11 ±0.02	0.09 ±0.01	0.09 ±0.03	0.03 ±0.01	0.08 ±0.02	0.05 ±0.02	0.09 ±0.02	0.19 ±0.03	0.10 ±0.02
29	***β*-Bourbonene**	Herbal, woody, floral, balsamic	1396	1396	0.03 ±0.01	0.04 ±0.01	0.08 ±0.02	0.07 ±0.02	0.06 ±0.01	0.07 ±0.01	0.06 ±0.01	0.15 ±0.03	0.06 ±0.01	0.06 ±0.0	0.06 ±0.01	0.06 ±0.02	0.06 ±0.02	0.08 ±0.02
30	***trans*-*β*-Caryophyllene**	Softly spicy, woody	1435	1444	1.06 ±0.45	1.99 ±0.38	4.44 ±0.86	0.67 ±0.09	0.27 ±0.04	0.46 ±0.09	1.01 ±0.12	2.11 ±0.13	1.19 ±0.03	10.19 ±0.91	0.13 ±0.03	0.24 ±0.07	0.19 ±0.06	0.11 ±0.03
31	**Germacrene-D**	Strong sharp aroma with herbal, basil like notes	1495	1484	0.05 ±0.02	0.08 ±0.02	0.21 ±0.05	0.05 ±0.02	0.02 ±0.01	0.05 ±0.01	0.04 ±.01	0.08 ±0.02	0.05 ±0.01	0.40 ±0.05	0.02 ±0.01	0.03 ±0.01	0.02 ±0.01	0.04 ±0.01
32	***α*-Humulene**	Spicy, earthy, and woody	1471	1477	0.12 ±0.04	0.04 ±0.01	0.04 ±0.01	0.18 ±0.03	0.04 ±0.01	0.04 ±0.01	0.04 ±0.01	0.04 ±0.01	0.03 ±0.01	0.05 ±0.01	0.02 ±0.01	0.02 ±0.01	<LOQ	0.13 ±0.04
33	***α*-Caryophyllene**	Sweet, woody-spice	1486	1478	0.04 ±0.01	0.05 ±0.01	0.20 ±0.02	0.03 ±0.01	0.03 ±0.01	0.05 ±0.01	<LOQ	0.04 ±0.01	0.03 ±0.01	0.02 ±0.01	<LOQ	<LOQ	<LOQ	<LOQ
34	***g*-Muurolene**	Herbal,woody-spice	1486	1479	0.04 ±0.01	0.05 ±0.01	0.08 ±0.02	0.03 ±0.01	0.06 ±0.02	0.11 ±0.04	0.12 ±0.03	<LOQ	<LOQ	0.03 ±0.01	0.04 ±0.01	<LOQ	0.04 ±0.01	0.03 ±0.01
35	**Pentadecane (C15)**	Odorless	1500	1500	0.09 ±0.02	0.11 ±0.02	0.18 ±0.03	0.09 ±0.02	0.14 ±0.02	0.39 ±0.05	0.10 ±0.02	0.14 ±0.02	0.06 ±0.01	0.06 ±0.02	0.12 ±0.02	0.09 ±0.02	0.10 ±0.03	0.08 ±0.02
36	***α*-Muurolene+Benzyl tiglate**	Woody/Earthy, mushroom, green	1509	1526	0.06 ±0.01	0.11 ±0.02	0.24 ±0.02	0.05 ±0.01	0.03 ±0.01	0.08 ±0.02	0.04 ±0.01	0.02 ±0.01	0.05 ±0.01	0.15 ±0.0	0.07 ±0.02	0.05 ±0.01	0.02 ±0.01	0.05 ±0.01
37	***δ*-Cadinene**	Mild Spicy, dry woody, forest	1529	1522	0.15 ±0.05	0.23 ±0.06	0.50 ±0.05	0.14 ±0.04	0.23 ±0.04	0.46 ±0.06	<LOQ	0.03 ±0.01	0.09 ±0.02	0.03 ±0.01	0.03 ±0.01	0.11 ±0.03	0.03 ±0.01	0.10 ±0.08
38	**Heptadecene (C17:1)**	Odorless	1680	1677	0.17 ±0.02	0.25 ±0.04	0.75 ±0.07	0.14 ±0.03	0.309 ±0.05	1.35 ±0.08	0.22 ±0.03	0.36 ±0.04	0.17 ±0.04	0.11 ±0.07	0.17 ±0.05	0.33 ±0.05	0.18 ±0.03	0.19 ±0.04
39	**Heptadecane (C17)**	Odorless	1700	1700	0.28 ±0.05	0.29 ±0.05	1.40 ±0.09	0.19 ±0.03	0.41 ±0.05	1.83 ±0.05	0.18 ±0.02	0.32 ±0.03	0.21 ±0.02	0.22 ±0.09	0.21 ±0.04	0.28 ±0.04	0.18 ±0.02	0.23 ±0.04
40	***trans,trans*-*β*-Farnesol**	Very mild and delicate green floral odour with typical lily of the valley character	1720	1713	<LOQ	<LOQ	<LOQ	<LOQ	<LOQ	<LOQ	<LOQ	<LOQ	<LOQ	<LOQ	0.02 ±0.01	<LOQ	0.03 ±0.01	<LOQ
41	**Nonadecene (C19:1)**	Odorless	1863	1874	0.37 ±0.13	0.71 ±0.11	3.2 ±0.21	0.23 ±0.04	1.09 ±0.12	3.21 ±0.21	0.45 ±0.08	0.91 ±0.08	0.40 ±0.04	0.43 ±0.07	0.63 ±0.03	0.84 ±0.06	0.45 ±0.03	0.68 ±0.03
42	**Nonadecane (C19)**	Odorless	1900	1900	1.11 ±0.28	2.16 ±0.18	10.95 ±0.91	0.91 ±0.16	2.19 ±0.18	8.72 ±0.34	0.56 ±0.07	2.15 ±0.12	0.82 ±0.09	1.63 ±0.1	2.06 ±0.09	1.91 ±0.09	0.91 ±0.04	1.61 ±0.05
43	**Eicosane (C20)**	Odorless	2000	2000	<LOQ	0.04 ±0.01	0.37 ±0.07	0.09 ±0.02	0.07 ±0.02	0.25 ±0.05	<LOQ	0.04 ±0.01	<LOQ	<LOQ	0.05 ±0.1	0.03 ±0.00	0.04 ±0.02	0.06 ±0.02
44	**Heneicosane (C21)**	Odorless	2100	2100	<LOQ	0.05 ±0.02	1.4 ±0.04	0.01 ±0.01	0.12 ±0.05	0.87 ±0.05	<LOQ	0.09 ±0.02	<LOQ	0.02 ±0.01	0.22 ±0.04	0.12 ±0.02	0.08 ±0.02	0.05 ±0.01
45	**Tricosane (C23)**	Odorless	2300	2300	<LOQ	<LOQ	<LOQ	<LOQ	<LOQ	<LOQ	<LOQ	<LOQ	<LOQ	<LOQ	<LOQ	<LOQ	<LOQ	<LOQ
46	**Pentacosane (C25)**	Odorless	2500	2500	<LOQ	<LOQ	<LOQ	<LOQ	<LOQ	<LOQ	<LOQ	<LOQ	<LOQ	<LOQ	<LOQ	<LOQ	<LOQ	<LOQ
47	**Heptacosane (C27)**	Odorless	2700	2700	<LOQ	<LOQ	<LOQ	<LOQ	<LOQ	<LOQ	<LOQ	<LOQ	<LOQ	<LOQ	<LOQ	<LOQ	<LOQ	<LOQ

Table S1. VOCs profile, observed in *R. alba* L. fresh blossom by HS-SPME-GC, Mean (in rel. %)±SD.
Legend: W-whole blossom, P-petals, the numbers 30, 45, 60 indicate the SPME performed at 30, 45, 60°C, respectively.

No	Compound	Odour detection treshold, in water (ppb)*	CAS number
1	α-Pinene	6	80-56-8
2	Benzaldehyde	350-3500	100-52-7
3	β-Pinene/β-Myrcene	140/13-15	80-56-8/ 123-35-3
4	Benzyl alcohol	10 000	100-51-6
5	Phenyl acetaldehyde	4	122-78-1
6	Linalool	6	78-70-6
7	*cis*-Rose oxide	0.5	3033-23-6
8	2-Phenyl ethyl alcohol	750-1100	60-12-8
9	*trans*- Rose oxide	160	876-18-6
10	β-Citronellol + Nerol	40/300	106-22-9 + 106-25-2
11	*Z*-Citral (Neral)	30	106-26-3
12	Geraniol	40-75	106-24-1
13	*E*-Citral (Geranial)	32	141-27-5
14	Geranyl acetate	9	105-87-3
15	β-Damascone	1.5	23726-91-2
16	*trans*-β-Caryophyllene	64	87-44-5
17	Pentadecane (C15)	-	629-62-9
18	Heptadecene (C17:1)	-	6765-39-5
19	Heptadecane (C17)	-	629-78-7
20	Nonadecene (C19:1)	-	18435-45-5
21	Nonadecane (C19)	-	629-92-5

Table S2. Main components, observed in *R. alba* L. fresh blossom by HS-SPME-GC, with CAS-numbers and OD thresholds, in water (ppb), according to * http://www.leffingwell.com/odorthre.htm

Method	SPME conditions		
	Conditioning		Extraction
	Temperature, ^0C	Duration, min	Duration, min
M2	60	15	45
M21	60	15	30
M22	60	15	60
M23	60	30	45
M24	60	30	30
M25	60	30	60
M26	60	45	30
M27	60	45	60
M28	60	45	45
M5	45	15	30
M51	45	15	45
M52	45	15	60
M53	45	30	30
M54	45	30	45
M55	45	30	60
M56	45	45	30
M57	45	45	45
M58	45	45	60
M8	30	30	30
M81	30	30	45
M82	30	30	60
M83	30	45	30
M84	30	45	45
M85	30	45	60
M86	30	15	30
M87	30	15	45
M88	30	15	60

Table S3. SPME experimental conditions used for the method optimization.
